# Genome-Wide Identification, Expression and Interaction Analysis of GmSnRK2 and Type A PP2C Genes in Response to Abscisic Acid Treatment and Drought Stress in Soybean Plant

**DOI:** 10.3390/ijms232113166

**Published:** 2022-10-29

**Authors:** Xinjie Shen, Hong Nan, Yuzhuang Jiang, Yujia Zhou, Xuejun Pan

**Affiliations:** College of Agriculture, Guizhou University, Guiyang 550025, China

**Keywords:** soybean, SnRK2, PP2C-A, abscisic acid, drought stress, protein interaction

## Abstract

As a typical ancient tetraploid, soybean (*Glycine max*) is an important oil crop species and plays a crucial role in supplying edible oil, plant protein and animal fodder worldwide. As global warming intensifies, the yield of soybean in the field is often strongly restricted by drought stress. SNF1-related protein kinase 2 (SnRK2) and type A protein phosphatase 2C (PP2C-A) family members are core components of the abscisic acid (ABA) signal transduction pathway in plants and have been suggested to play important roles in increasing plant tolerance to drought stress, but genetic information supporting this idea is still lacking in soybean. Here, we cloned the *GmSnRK2s* and *GmPP2C-A* family genes from the reference genome of Williams 82 soybean. The results showed that the expression patterns of *GmSnRK2s* and *GmPP2C-As* are spatiotemporally distinct. The expression of *GmSnRK2s* in response to ABA and drought signals is not strictly the same as that of *Arabidopsis SnRK2* homologous genes. Moreover, our results indicated that the duplicate pairs of *GmSnRK2s* and *GmPP2C-As* have similar expression patterns, *cis*-elements and relationships. *GmSnRK2.2* may have a distinct function in the drought-mediated ABA signaling pathway. Furthermore, the results of yeast two-hybrid (Y2H) assays between GmSnRK2s and GmPP2C-As revealed that *GmSnRK2.17*, *GmSnRK2.18*, *GmSnRK2.22*, *GmPP2C5*, *GmPP2C7*, *GmPP2C10* and *GmPP2C17* may play central roles in the crosstalk among ABA signals in response to drought stress. Furthermore, *GmPP2C-A*s and *GmSnRK*s were targeted by miRNA and validated by degradome sequencing, which may play multiple roles in the crosstalk between ABA and drought signals and other stress signals. Taken together, these results indicate that GmSnRK2s and GmPP2C-As may play a variety of roles in the drought-mediated ABA signaling pathway.

## 1. Introduction

Plant hormones are a group of small organic signaling molecules that act as bridges to mediate the communication and subsequent responses of plants, both to endogenous molecular signal transduction and environmental adaptation. Along with the well-known six plant hormones, abscisic acid (ABA) has been suggested to play an essential role throughout the entire life of plants, including roles in seed dormancy and germination, bud dormancy, root development, stomatal movement, leaf senescence, vegetative development, flowering and fruit ripening [1,2,3]. In addition to its roles in various aspects of plant growth and development, ABA also serves as a key endogenous messenger in adapting to abiotic and biotic stress responses in plants [4]. Many studies have suggested that ABA plays essential roles in the response to multiple abiotic stresses, including drought, cold, salinity and heat stress [5,6,7,8]. Drought has been and continues to be a severe ecological problem worldwide, and strongly impacts crop yields and food security for humans [9]. ABA is the major stress-responsive hormone produced after drought signals are perceived. Plants have evolved sophisticated interconnected signaling pathways to overcome drought stress. ABA is the key player in the plant response to drought stress. ABA is synthesized and accumulates in plant leaves, which can then promote stomatal closure, reduce transpiration and ensure water balance [10]. In the roots, ABA can promote root elongation so that the roots reach deep soil to obtain more water under drought conditions [11]. Moreover, in the roots, ABA can suppress the synthesis of reactive oxygen species (ROS) and ethylene, which act as root growth inhibitors to increase root length and density, specifically when the soil cannot hold water or when water is available deeper in the soil [1,12]. Furthermore, ABA can activate numerous cellular responses in plants through a series of signal transduction networks and pathways that interact with other drought-related phytohormones. ABA can also inhibit auxin signaling repressors (*AXR3/AA17*) and increase the expression of *DR5* and *IAA2*, both of which act as auxin reporters to activate auxin signaling to transport auxin towards elongating root cells; in turn, auxin promotes root growth to take up deep water under drought conditions [13,14]. In addition to its interaction with auxin, ABA has also been reported to undergo negative crosstalk with gibberellin (GA), which can downregulate the DELLA protein PRO and upregulate ABA transport with *AIT1.1*, ultimately promoting guard cell responses under drought conditions [15]. Thus, ABA plays a vital role in the plant response to drought stress.

**Figure 1:**
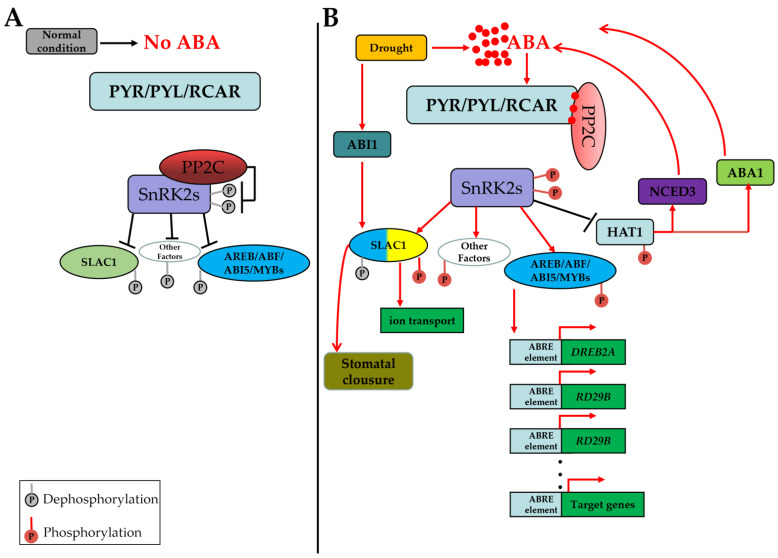
ABA signal transduction model. (**A**) Normal condition. (**B**) Under drought condition. Black phosphate represent Dephosphorylation; Red phosphate represent phosphorylation.

When plants are under drought conditions, the PYR/PYL/RCAR ABA receptor protein family members can perceive drought signals from the environment and then trigger ABA signal transduction through protein phosphorylation reactions in cells [16]. It has been well demonstrated that PYR/PYL/RACR and members of the type 2C protein phosphatase (PP2C) and SNF1-related kinase protein kinase 2 (SnRK2) families are core ABA signaling components. The classic method of ABA signal transduction can be described as follows: in the absence of ABA, the activity of SnRK2s is inhibited by physiological interaction with type A PP2Cs (PP2C-A) via the dephosphorylation of multiple Ser/Thr residues in an activation loop (Figure 1A); in the presence of ABA, the structure of the PYR/PYL/RCAR ABA receptors changes in response to binding ABA molecules, which enables the interaction of ABA receptors with PP2Cs and leads to suppression of PP2C-mediated dephosphorylation of SnRK2s (Figure 1B); and finally, SnRK2s are released from PP2C inhibition and are able to activate their downstream targets, such as ABA response element-binding factors (ABFs), ABA-INSENSITIVE (ABI) transcription factors, MYB transcription factors and ABA-responsive element-binding proteins (AREBs) [2,7,16,17,18]. In the model plant species *Arabidopsis*, the *SnRK2* family can be divided into three groups [19]. Group I members (*AtSnRK2.1*, *AtSnRK2.4*, *AtSnRK2.5*, *AtSnRK2.9*, and *AtSnRK2.10*) are mainly involved in the response to osmotic stress and also increase drought tolerance, but do not respond to ABA signaling [19]. Group II members (*AtSnRK2.7* and *AtSnRK2.8*) can respond to salt stress and weakly respond to ABA signals, and they can also improve drought tolerance [20,21]. Group III members (*AtSnRK2.2*, *AtSnRK2.3*, and *AtSnRK2.6*) have been proven to regulate ABA-induced stomatal movement and modulate seed germination and primary root growth [22,23]. The group A-type PP2Cs of *Arabidopsis* have been proven to act as central negative modulators of the ABA signaling pathway [24].

The identification of the ABA signal transduction core unit has greatly promoted advances in understanding plant ABA signal transduction under drought conditions, the utilization of ABA signaling mechanisms for manipulation of plant physiological structure responses, as well as for enhancement of the drought tolerance and water use efficiency of plants. The ABA signal transduction pathway can be activated in plants to regulate the expression of *ABI1* and *OST1* to generate H_2_O_2_, the secondary messengers, which can mediate stomatal closure to balance plant cell water contents under drought conditions [3,25]. A recent study showed that SnRK2.3 could phosphorylate and inactivate HD-ZIP (HAT1), potentially suppressing the expression of both *ABA3* and *NCED3*, which negatively regulate ABA biosynthesis [26]. This leads to increased ABA biosynthesis, amplifying the ABA signal which increases plant resistance to drought stress. These results indicated that the core plant ABA signal transduction pathway is not a one-way and loop-locked signaling pathway but, rather, is a self-feedback and open-ended pathway [10,16].

Plant growth and stress responses are also carefully controlled by both the activity and the abundance of PP2Cs. Although stress induces ABA inactivation of PP2Cs, releasing SnRK2s and initiating the ABA signal transduction pathway, the expression levels of *PP2Cs* are actually upregulated by the ABA signal through the action of certain regulatory factors, such as ABFs; this creates a negative feedback loop control mechanism, with appropriate homeostatic levels maintained in order to desensitize the plant to high ABA levels [26,27]. Recent studies have suggested that PP2C-As are the central negative regulators of the ABA signal transduction pathway and can be induced in response to ABA and drought stress [28,29]. In addition to the interactions of some PP2C-As with SnRK2s which negatively regulate the ABA signaling pathway, the activity of some PP2C-As can also be enhanced by direct interaction with EAR1 proteins [30], indicating that PP2C-As also function under an open-loop and feedback regulatory mechanism to maintain their activity (Figure 1B). The PPC-A member ABI1 can directly dephosphorylate the N-terminus of SLAC1 to affect SLAC1-mediated stomatal closure under drought conditions [31,32].

Soybean (*Glycine max* L. *Merr.*) is an important source of protein and cooking oil for humans and fodder for animal husbandry. As global warming intensifies, soil drought is becoming increasingly severe and has an increasing impact on soybean yields. In plants, the ABA signal transduction pathway plays a central role in the response to drought stress. Although the SnRK2s and PP2Cs core ABA signaling components have been extensively studied in model plant species and in some crop plant species, the expression patterns of Mer *PP2C-As* and *SnRK2s* in different soybean tissues under ABA and drought treatment remain unclear. Moreover, the ABA- and drought-related interactions between PP2C-As and SnRK2s are poorly understood. In this article, we identified and evaluated the expression members of the *PP2C-As* and *SnRK2* families from the soybean genome under ABA and drought treatment. Moreover, we analyzed the possible interactions between PP2C-As and SnRK2s, both of which function in response to ABA and drought stress. This study presents the results of the first genome-wide analysis of the PP2C-A and SnRK gene families in response to ABA and drought treatment in soybean. Taken together, these results will broaden our insight into the roles of *PP2C-A* and *SnRK2* genes in the drought-induced ABA signal transduction pathway and provide a foundation for further discoveries of new interaction factors and molecular regulatory mechanisms.

## 2. Results

### 2.1. Identification and Sequence Analysis of GmPP2C-A and GmSnRK2 Genes

**Figure 2:**
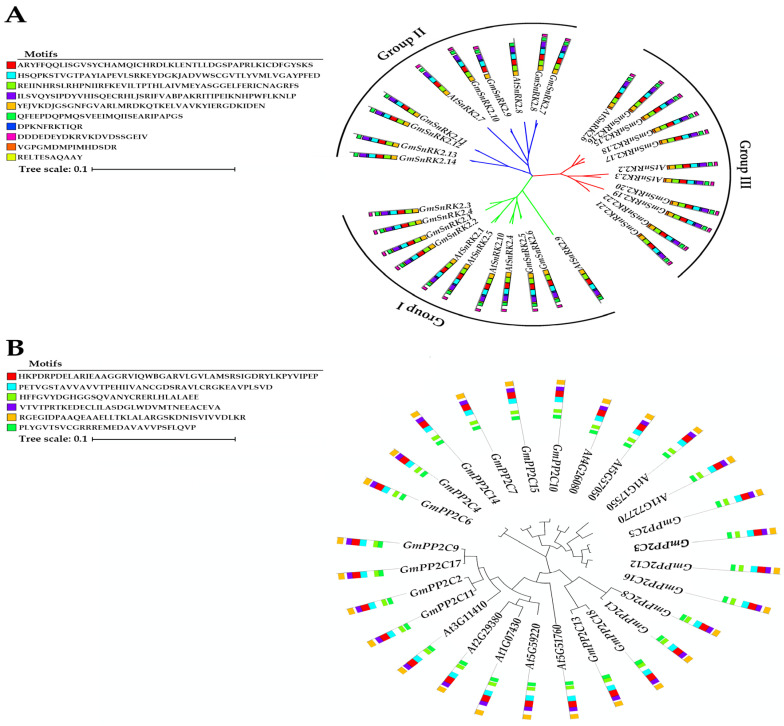
Phylogenetic analysis and distribution of the conserved motifs in *GmSnRK2s* and *GmPP2C-As*. (**A**) Phylogenetic relationships and conserved motifs in *GmSnRK*s and *AtSnRK2s.* (**B**) Phylogenetic relationships and conserved motifs in *GmPP2C-As* and *AtSnRK2s*. The ML phylogenetic tree shown was constructed via full-length protein sequences and with 1000 bootstraps. The conserved motifs were detected using MEME software and are represented by coloured boxes. *Gm* = *G. max* and *At* = *A. thaliana*.

Gene-specific primers were used to perform gene-specific PCR, resulting in the isolation of the full-length coding sequences of eighteen *GmPP2Cs* (*GmPP2C1*- *GmPP2C18*) and twenty-two *GmSnRK2s* (*GmSnRK2.1-GmSnRK2.22*) from the cDNA of soybean leaves. Phylogenetic analysis, comparing the full coding DNA sequences (CDSs) of these genes with those of homologous genes in *Arabidopsis*, revealed that the soybean *PP2C* (Figure 2A) and *SnRK2* (Figure 2B) gene families could be clustered and grouped with the *Arabidopsis PP2C* and *SnRK2* gene families, respectively. Additionally, protein sequence analysis suggested that the members of the soybean PP2C-As gene family (*GmPP2C-As*) have multiple key functional domains, including PPM-type phosphatase domains, Mn^2+^-/Mg^2+^-binding sites, and several protein activation sites (Figure 2A and Figure S1). The results of the protein sequence analysis of SnRK2s revealed that SnRK2s also have many key functional domains, such as protein kinase domains, protein kinase A catalytic subunit domains, Ser/Thr-protein kinase activation sites, activation loops and ATP-binding sites (Figure 2B and Figure S2).

To explore the chemical and physical properties of the GmPP2C-A and GmSnRK2 proteins, the amino acid sequences encoded by these genes were profiled by ProtParam (http://web.expasy.org/protparam/, accessed on 16 July 2021) (Tables S1 and S2). The molecular weight (MW) of the GmPP2C-As and GmSnRK2s ranged from 26.6 to 60.82 KDa and from 25.64 to 41.08 KDa, respectively. The isoelectric point (pI) varied from 4.52 to 8.04 (GmPP2C-As) and from 4.69 to 8.33 (GmSnRK2s). These results suggested that, except for GmPP2C8 (pI: 7.55), GmPP2C13 (pI: 8.04) and GmSnRK2.12 (pI: 8.33), most members of the GmPP2C-As and GmSnRK2s were acidic. The instability index of the GmPP2C-A and GmSnRK2 proteins ranged from 37.34 to 67.40 and from 31.82 to 53.31, respectively, which suggested that the proteins of most members of these two gene families were unstable (aliphatic index > 40), except for GmPP2C16. Conversely, GmSnRK2.7, GmSnRK2.8, GmSnRK2.11, GmSnRK2.12, GmSnRK2.13, GmSnRK2.14, GmSnRK2.19, GmSnRK2.20, and GmSnRK2.22 were stable (aliphatic index < 40). The grand average of hydropathy (GRAVY) values of the GmPP2C-As and GmSnRK2s were all negative, indicating that the members of both gene families were hydrophilic and probably localized to the cytosol.

**Figure 3:**
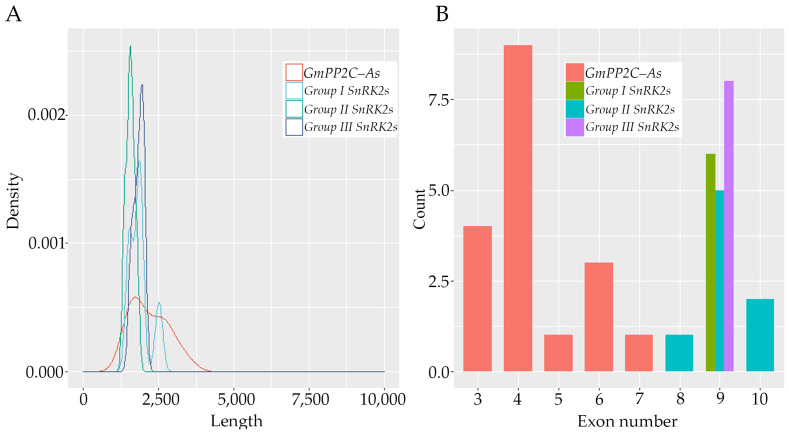
Length distribution (**A**) and exon numbers (**B**) of *GmPP2C-A* and *GmSnRK2* genes.

The nucleotide length and exon numbers were also analyzed. The length of the GmPP2C-A genes varied from 1434 to 3483 nt, with a mean of 1946 nt. The length of *SnRK2s* also ranged from 1353 to 2512 nt, with a median length of 1946 nt. Most genes in the group I, II, and III SnRK2s were shorter than 1900, 1700, and 2000 nt, respectively, with median lengths of 1863, 1560, and 1832 nt, respectively (Figure 3B). As described in Figure 3, the *GmPP2C-A* and *GmSnRK2* genes presented distinct intron-exon distributions. The exon number of the *GmPP2C-A* genes ranged from three to seven, and most of them had four exons. However, the data indicated that, in terms of exon-intron composition, there were no significant changes among the same group of *GmSnRK2* genes. For instance, all the group I, all the group III and the majority of the group II *GmSnRK2s* genes contained nine exons.

### 2.2. Analysis of Stress-Related Cis-Elements of GmPP2C-A and GmSnRK2 Genes

**Figure 4:**
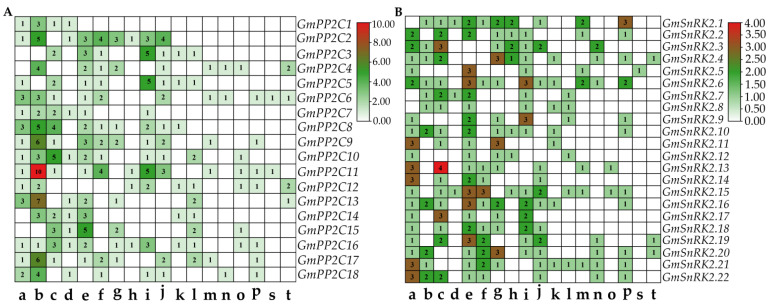
*Cis*-elements in the promoters of *GmPP2C-A* (**A**) and *GmSnRK2* (**B**) are related to hormone and stress responses. The bar indicates the number of *cis*-elements. The numbers 1, 2, 3, … represent the number of repeats of each *cis*-element, whereas 0 indicates absence of a particular *cis*-acting element. Letters a, b, c, … represent *cis*-elements. a: MBS (CAACTG, MYB-binding site involved in drought inducibility); b: ABRE (TACGTG, ABA response element); c: ARE (TGGTTT, anaerobic induce element); d: O_2_-site (GATGATGTGA, *cis*-acting regulatory element involved in zein metabolism regulation); e: TC-rich repeats (ATTTTATCCA, defence and stress response element); f: CGTCA-motif (CGTCA, MeJA response element); g: HSE (AAAAAATTAC, heat stress response element); h: P-box (CCTTTTG, GA response element); i: TCA-element (TCAGAAAAGG, salicylic acid response element); j: TGACG-motif (TGACG, MeJA response element); k: Box-W1 (TTGACC, fungal elicitor response element); l: GARE-motif (TCTGTTG, GA response element); m: long terminal repeat (LTR) (CCGAAA, low-temperature response element); n: TGA-element (AACGAC, auxin response element); o: WUN motif (AAATTTCCT, wound response element); p: ERE (ATTTCAAA, ethylene response element); s: CE3 (GATGCGTGTC, ABA and VP1 response element); t: CCAAT-box (CAACGG, MYBHv1-binding site).

To investigate and analyze the stress-related *cis*-element regulatory elements in the promoters of *GmPP2C-As* and *GmSnRK2s*, the genomic DNA sequences 2 kb upstream from the transcription initiation sites (TISs) were extracted and profiled via PlantCARE. The results obtained from the PlantCARE website showed that multiple stress-related regulatory elements are present within the promoter regions of these two families of genes (Figure 4A,B). For instance, thirteen *GmPP2Cs* (*GmPP2C1*, *GmPP2C2*, *GmPP2C5*, *GmPP2C6*, *GmPP2C7*, *GmPP2C8*, *GmPP2C9*, *GmPP2C10*, *GmPP2C11*, *GmPP2C12*, *GmPP2C13*, *GmPP2C16*, *GmPP2C17*, and *GmPP2C18*) and nineteen *GmSnRK2s* (*GmSnRK2.2*, *GmSnRK2.3*, *GmSnRK2.4*, *GmSnRK2.5*, and *GmSnRK2.6* of group I, *GmSnRK2.9*, *GmSnRK2.10*, *GmSnRK2.11*, *GmSnRK2.12*, *GmSnRK2.13*, and *GmSnRK2.14* of group II, and *GmSnRK2.15*, *GmSnRK2.16*, *GmSnRK2.17*, *GmSnRK2.18*, *GmSnRK2.19*, *GmSnRK2.20*, *GmSnRK2.21*, and *GmSnRK2.22* of group III) had one or more the MYB-binding sites (MBSs), which are involved in drought inducibility. Moreover, the following had one or more ABA-responsive elements (ABREs): sixteen *GmPP2Cs*, (*GmPP2C1*, *GmPP2C2*, *GmPP2C4*, *GmPP2C6*, *GmPP2C7*, *GmPP2C8*, *GmPP2C9*, *GmPP2C10*, *GmPP2C11*, *GmPP2C12*, *GmPP2C13*, *GmPP2C14*, *GmPP2C15*, *GmPP2C16*, *GmPP2C17*, and *GmPP2C18*); *GmSnRK2.1*, *GmSnRK2.3*, *GmSnRK2.4*, and *GmSnRK2.6* of group I; *GmSnRK2.7*, *GmSnRK2.8*, and *GmSnRK2.10* of group II; and *GmSnRK2.16*, *GmSnRK2.20*, *GmSnRK2.21*, and *GmSnRK2.22* of group III. In addition, several other stress-related plant hormone-responsive elements predicted to be ABREs, such as ARE/AuxRR core elements (auxin), CGTCA motif/TGA elements (methyl jasmonate (MeJA)), TCA elements (salicylic acid), P-boxes/GARE motifs/TATC boxes (GA) and ethylene response elements (EREs) (ethylene), were also present in the promoter regions of *GmPP2C-As* and *GmSnRK2s*. Furthermore, *cis*-elements involved in the response to abiotic and biotic stresses, including zein, heat, fungi and low temperature, were also found within the upstream regions of *GmPP2C-As* and *GmSnRK2s* (Figure 4A,B). Notably, *GmPP2C4*, *GmPP2C10*, *GmPP2C11*, *GmPP2C12*, *GmPP2C15*, and *GmPP2C16* as well as *GmSnRK2.13* and *GmSnRK2.15* contain *cis*-elements that function in response to wounding, which suggests that *GmPP2C-As* and *GmSnRK2s* might be associated with the response to biotic stress.

### 2.3. Chromosomal Distribution and Expansion Patterns of GmPP2C-A and GmSnRK2 Genes

**Figure 5:**
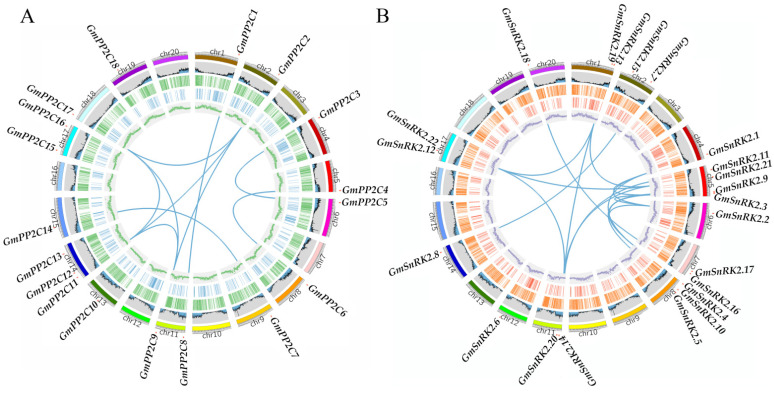
Chromosome distribution and synteny analysis of *GmPP2C-A* (**A**) and *GmSnRK2* (**B**) genes. Chromosomes 1–20 are illustrated in different colours. The approximate locations of all the genes are shown with short red lines. The blue curves indicate segmentally duplicated genes. The tracks from outside to inside represent the gene density, methylated region distributions of Dos-DMR (process of soybean domestication, Dos-DMR), methylated region distributions of Imp-DMR (in the improvement process, Imp-DMR), and GC density.

Soybean is a diploidized ancient tetraploid plant, and soybean cells contain 20 chromosomes. In this work, we analyzed the distribution of *GmPP2C-A* and *GmSnRK2* genes in the soybean genome. The results showed that the *GmPP2C-A* and *GmSnRK2* genes were unevenly distributed on chromosomes 14 and 12, respectively (Figure 5). Across the soybean chromosomes, chromosomes 14 and 5 contained the most *GmPP2C-As* (three genes) and *GmSnRK2s* (four genes), respectively (Figure 5). The other chromosomes had one to three *GmPP2C-A* and *GmSnRK2* genes. Chromosomes 3, 7, 10, 12, 16, and 20 carried no *GmPP2C-A* genes, and chromosomes 3, 9, 10, 13, 15, 16, 18, and 19 carried no *GmSnRK2* genes. Furthermore, we also analyzed the differentially methylated (DMRs) *GmPP2C-A* and *GmSnRK2* genes. The results showed that *GmPP2C-A* and *GmSnRK2* genes were located in a few DMR regions during soybean domestication and improvement (Figure 5). Moreover, except for *GmPP2C18*, *GmSnRK2.21* and *GmSnRK2.21*, all the *GmPP2C-A* and *GmSnRK* members were located in the high-GC-density regions in the soybean genome (Figure 5).

Gene duplication events are critical factors involved in the expansion of gene families during genome expansion. The results showed that 10 and 16 paralogous gene pairs of *GmPP2C-A* (Figure 5A and Table S3) and *GmSnRK2* (Figure 5B and Table S4) genes, respectively, were identified in soybean. To determine their duplication time, the nonsynonymous rate (*Ka*), synonymous rate (*Ks*) and the Ka/Ks ratios of paralogous gene pairs of *GmPP2C-As* and *GmSnRK2s* were analyzed (Tables S3 and S4). All the *Ka/Ks* ratios of *GmPP2C-As* and *GmSnRK2s* were less than 1, varying from 0.19 to 0.52 and from 0.02 to 0.24, respectively. These results suggested that the paralogous gene pairs of *GmPP2C-As* and *GmSnRK2s* were strongly selected during the expansion of the soybean genome. Moreover, we also estimated the duplication time. The duplication time of *GmPP2C-As* and *GmSnRK2s* varied from 7.5 to 80.2 million years ago (MYA) and from 6.1 to 46.4 MYA, respectively. These results indicated that all the duplication events of the two gene families occurred in the latest whole-genome duplication of soybean.

### 2.4. Gene Ontology (GO) Annotation

**Figure 6:**
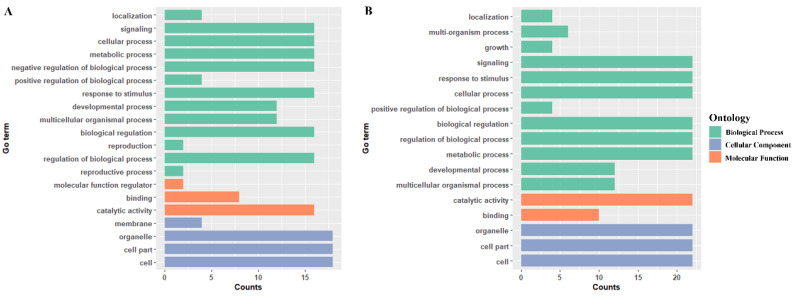
GO enrichment analysis. (**A**) GO enrichment of *GmPP2C-A* genes. (**B**) GO enrichment of *GmSnRK2* genes. According to the secondary terminology, the annotation results are divided into three ontology categories and distinguished by different colors.

In order to further predict the functions of *GmPP2C-A* and *GmSnRK2* genes, GO (gene ontology) annotation analyses were performed. A total of 22 distinct functional groups were determined: 15 involved in biological processes, 4 involved in cellular components and 3 involved in molecular functions (Figure 6). In biological processes, GO classifications of ‘reproductive process’, and ‘growth’ and ‘multi-organism process’ were specifically attributed in *GmPP2C_GroupA* and *GmSnRK2* genes, respectively. Moreover, *GmPP2C_GroupA* genes could perform negative and positive regulation of biological processes, while *GmSnRK2* members could only undertake positive regulation of biological processes. As for genes in the cellular component part, 18 *GmPP2C-A* genes were annotated with ‘membrane’, ‘organelle’, ‘cell part’, and ‘cell’, while 22 *GmSnRK2* genes were associated with ‘organelle’, ‘cell part’, and ‘cell’. Under the molecular function term, 16, 8, and 2 genes were annotated with ‘catalytic activity’, ‘binding’, and ‘molecular function regulator’, respectively, while only 22 and 10 genes were annotated to have the designations ‘catalytic activity’ and ‘binding’. All these results indicate the multiple functions of *GmPP2C-A* and *GmSnRK2* genes.

### 2.5. Prediction of GmPP2C-A and GmSnRK2 Genes Targeted by miRNAs

**Figure 7:**
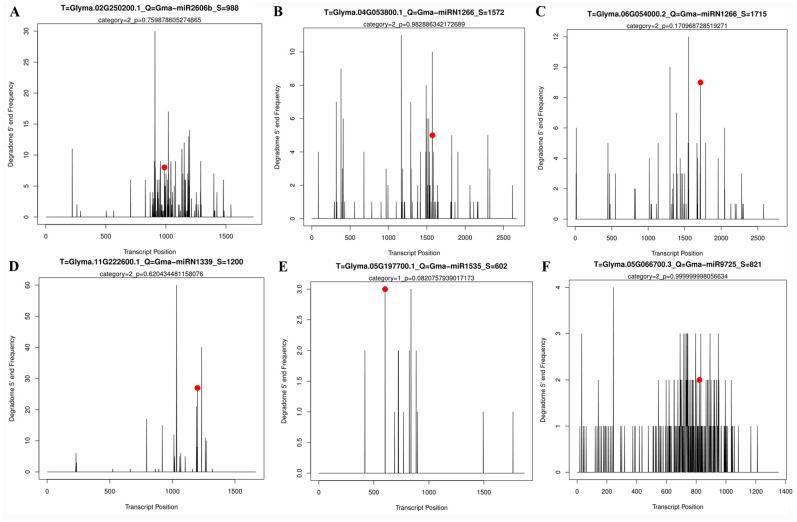
Post-transcriptional regulation of *GmPP2C-A* and *GmSnRK2* by degradome sequencing. (**A**) Cleavage features in GmPP2C2 by Gma-miR2606b. (**B**) Cleavage features in GmPP2C3 by Gma-miR1266. (**C**) Cleavage features in GmPP2C5 by Gma-miR1266. (**D**) Cleavage features in GmPP2C9 by Gma-miRN1339. (**E**) Cleavage features in GmSnRK2.3 by Gma-miR1535. (**F**) Cleavage features in GmSnRK2.11 by Gma-miR9725. Vertical axes display the read abundance and the horizontal axes display the precursor position by base pair. The black vertical bars show the read abundance for the corresponding precursor base. The red dots mean the cleavage nucleotide positions on the target genes. The corresponding *p* values are shown above the plots.

In order to obtain a deep understanding of the functional roles of *GmPP2C-A* and *GmSnRK2* genes in soybean, we evaluated the potential regulation of these two gene families by miRNA targets prediction. The results showed that four miRNA-GmPP2C-As pairs and two miRNA-GmSnRK pairs were validated by degradome sequencing. These pairs included Gma-miR2606b: GmPP2C2 (Glyma.02G250200), Gma-miRN1266: GmPP2C3 (Glyma.04G053800), Gma-miRN1266: GmPP2C5 (Glyma.06G054000), Gma-miRN1339: GmPP2C9 (Glyma.11g222600), Gma-miR1535: GmSnRK2.3 (Glyma.05g197700), and Gma-miR9725: GmSnRK2.11 (Glyma.05G066700) (Figure 7). As illustrated in Figure 6, the duplicated gene pair GmPP2C3 and GmPP2C5 transcripts were identified as both being cleaved by Gma-miRN1266, while another duplicated gene pair (GmPP2C2 and GmPP2C9) was cleaved by Gma-miR2606b and Gma-miRN1339, respectively.

### 2.6. Expression Profiles of GmPP2C-As in Response to Exogenous ABA Treatment and Drought Stress

ABA has been proven to play a crucial role in plants in response to diverse environmental stresses. Along with its role in response to these environmental stresses, ABA has been demonstrated to act as a molecular signal in the drought signal transduction pathway. To explore the expression patterns of *GmPP2C-As* in response to drought stress and ABA, we measured the expression levels of *GmPP2C-As* by qRT-PCR in response to exogenous ABA and drought stress treatment.

**Figure 8:**
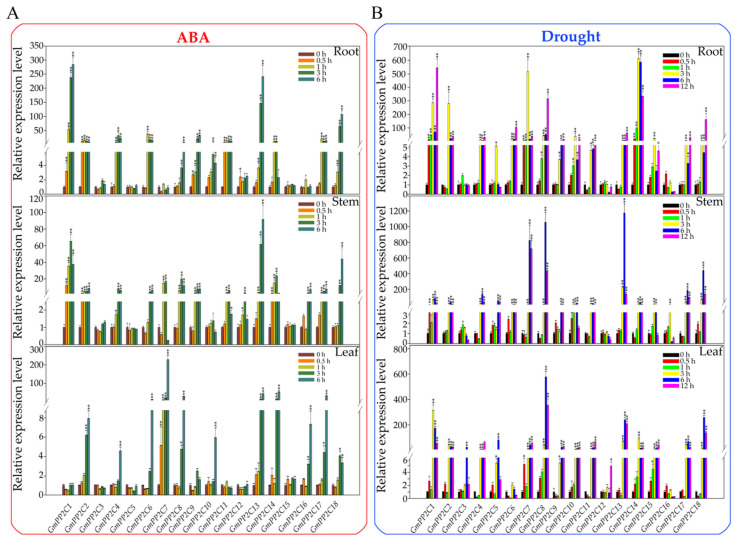
Expression analysis of *GmPP2C-As* under ABA and drought treatments. (**A**) Expression analysis of *GmPP2C-As* in the roots, stems and leaves of soybean under ABA treatment. (**B**) Expression analysis of *GmPP2C-As* in the roots, stems and leaves of soybean under drought treatment. The expression levels in the roots, stems and leaves before treatment (0 h) were used as controls and were assigned a value of 1. *GmSKIP* was used as an internal reference. Each point represents the mean value ± SE of three independent experiments performed in triplicate. The differences were statistically assessed using Student’s *t test* (**, *p* < 0.01; *, *p* < 0.05). The error bars in A and B represent the SEs of three replicates.

The expression levels of *GmPP2C-As* were measured in the root and leaf tissues at five time points: 0, 0.5, 1, 3 and 6 h. The results showed that all *GmPP2C-As*, except for *GmPP2C3*, were significantly upregulated at four time points (0.5, 1, 3 and 6 h) under ABA treatment compared to those in the control roots (Figure 8A). Similar to the roots, the expression levels of all *GmPP2C-As* (excluding *GmPP2C3*) were upregulated significantly in the stems under ABA treatment (Figure 8A). In contrast to those in the roots and stems, the expression levels of *GmPP2C-As* exhibited different patterns in the leaves. In terms of expression, of the 18 *GmPP2C* genes, *GmPP2C2*, *GmPP2C4*, *GmPP2C6*, *GmPP2C7*, *GmPP2C8*, *GmPP2C10*, *GmPP2C13*, *GmPP2C14*, *GmPP2C16*, *GmPP2C17* and *GmPP2C18* were upregulated in response to ABA treatment, while *GmPP2C3* and *GmPP2C11* were downregulated (Figure 8A). Taken together, these results suggested that most of the group A-type *GmPP2C* family genes could respond to ABA treatment but exhibited different expression patterns in different tissues of soybean seedlings.

When plants are under drought stress, ABA is always synthesized, increasing resistance to the stress conditions. In the present study, we also measured the expression levels of *GmPP2C-As* family genes in response to drought stress at five time points (0.5, 1, 3, 6 and 12 h) in the roots, stems and leaves. As shown in Figure 8B, following drought stress, *GmPP2C1*, *GmPP2C2*, *GmPP2C4*, *GmPP2C6*, *GmPP2C7*, *GmPP2C8*, *GmPP2C9*, *GmPP2C10*, *GmPP2C11*, *GmPP2C13*, *GmPP2C14*, *GmPP2C15*, *GmPP2C17* and *GmPP2C18* were upregulated significantly during drought stress compared to those of the control treatment (Figure 8B). Similar to ABA treatment, *GmPP2C-As* had similar expression patterns in the roots and stems under drought treatment (Figure 8B). In contrast to the results under ABA treatment, the expression levels of *GmPP2C-As* under drought treatment were similar in the leaves compared to that in the roots and stems (Figure 8B).

Taken together, these results suggested that most *GmPP2C-As* could respond to exogenous ABA and drought treatment, but exhibit different expression patterns in different tissues.

### 2.7. Expression Profiles of GmSnRK2s in Response to Exogenous ABA Treatment and Drought Stress

To expand our knowledge of the molecular function of *GmSnRK2* genes in response to ABA treatment and drought stress, we measured their expression levels under exogenous ABA treatment and drought stress by qPCR in the roots, stems and leaves. In the roots, after ABA treatment, all *GmSnRK2s* except *GmSnRK2.3*, *GmSnRK2.5*, *GmSnRK2.6*, *GmSnRK2.9*, *GmSnRK2.11*, *GmSnRK2.12*, *GmSnRK2.13*, *GmSnRK2.14*, *GmSnRK2.15*, *GmSnRK2.16* and *GmSnRK2.17* showed differential expression during the time course of the treatment. The expression trends of *GmSnRK2s* in the stems were similar to those in the roots (Figure 8A). The expression levels of *GmSnRK2s* in the leaves were slightly different from those in the roots and stems, and the expression levels of *GmSnRK2.18*, *GmSnRK2.20* and *GmSnRK2.22* in the leaves were significantly higher than those in the roots and stems.

**Figure 9:**
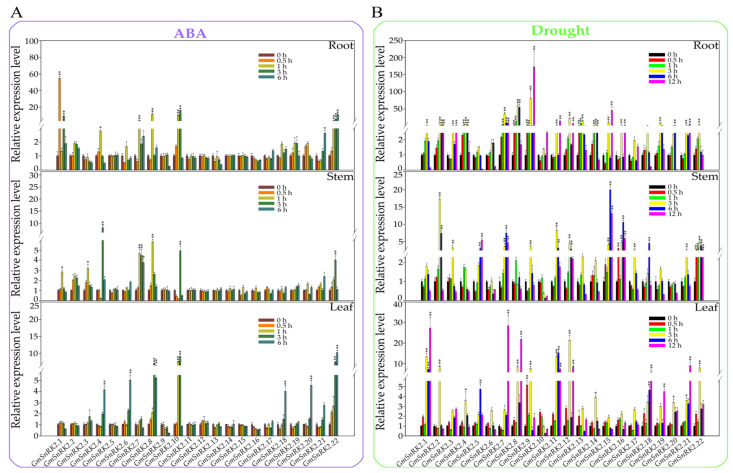
Expression analysis of *GmSnRK2s* under ABA and drought treatments. (**A**) Expression analysis of the *GmSnRK2s* in the roots, stems and leaves of soybean under ABA treatment. (**B**) Expression analysis of the *GmSnRK2s* in the roots, stems and leaves of soybean under drought treatment. The expression levels in roots, stems and leaves before treatment (0 h) were used as controls and were assigned a value of 1. *GmSKIP* was used as an internal reference. Each point represents the mean value ± SE of three independent experiments performed in triplicate. The differences were statistically assessed using Student’s *t* test (**, *p* < 0.01; *, *p* < 0.05). The error bars in A and B represent the SEs of three replicates.

After drought stress treatment, most of the *GmSnRK2* genes responded to drought stress (Figure 9B). The expression trends of *GmSnRK2.1*, *GmSnRK2.8*, *GmSnRK2.9*, *GmSnRK2.11*, *GmSnRK2.12*, *GmSnRK2.18* and *GmSnRK2.21* were gradually upregulated in roots, stems and leaves. Among these *GmSnRK2* genes, the expression patterns of *GmSnRK2.8* and *GmSnRK2.22* were similar to those observed under exogenous ABA treatment, which suggested that *GmSnRK2.8* and *GmSnRK2.22* might play roles in the ABA-mediated drought stress response. These results suggested that the *GmSnRK2* genes might play a variety of roles in the soybean ABA signaling pathway and response to drought stress.

### 2.8. Soybean GmPP2C-As Interact with GmSnRK2s

**Figure 10:**
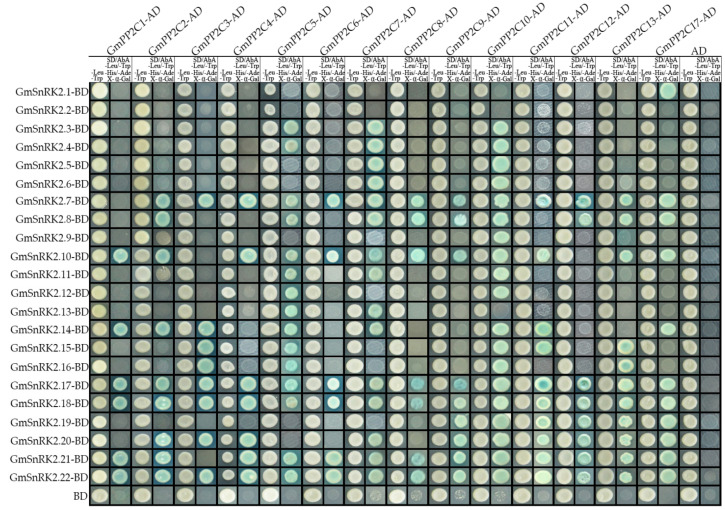
Physical interactions between GmPP2C-As and GmSnRK2s, determined using a Y2H system. A pGADT7 vector was used to express GmPP2C-As, and a pGBKT7 vector was used to express GmSnRK2s. The *GmPP2C-AD* and *GmSnRK2-BD* constructs were then co-transformed into yeast and selected on the basis of the ability of the yeast to grow on media lacking leucine (Leu) and tryptophan (Trp). The interaction was indicated by the ability of yeast to grow on selective media that included aureobasidin A (AbA) and X–*α*–Gal, but lacked Leu, Trp, histidine (His), and adenine (Ade). The interactions of the PacSnRK2-AD constructs with pGBKT7 were used as controls to test for yeast self-activation.

In this work, we used a Y2H yeast system to analyze the interactions between GmPP2C-As and GmSnRK2s. Based on the results of the transcriptional activation effect of GmPP2C-As and GmSnRK2s, we tested the interactions between GmPP2C-As and GmSnRK2s (excluding GmPP2C14, GmPP2C15, GmPP2C16 and GmPP2C18). As shown in Figure 10, GmSnRK2s exhibited complex interactions with GmPP2C-As. GmSnRK2.7, GmSnRK2.8, GmSnRK2.10, GmSnRK2.14, GmSnRK2.17, GmSnRK2.18, GmSnRK2.20 and GmSnRK2.22 could interact with most of the GmPP2C-As, suggesting that, in conjunction with GmPP2C-As, these *GmSnRK2s* may play a basic role in the ABA signal transduction pathway. Other GmSnRK2s, such as group I GmSnRK2s (GmSnRK2.1-2.6), some group II GmSnRK2s (GmSnRK2.9, GmSnRK2.11, GmSnRK2.12, and GmSnRK2.13) and GmSnRK2.16, could interact with one to four GmPP2C-As. Notably, GmSnRK2.1 was found to interact with none of the GmPP2C-A genes: this indicated that GmSnRK2.1 may not respond to ABA and drought signals. Furthermore, all the tested GmPP2C-As could interact with most of the Group III GmSnRK2s (GmSnRK2.15-2.22), suggesting that soybean GmPP2C-As are involved in the ABA signal transduction pathway.

## 3. Discussion

ABA has been proven to play a crucial role in plants in response to various environmental stresses [1,2]. Along with its role in the response to these environmental stresses, ABA has been demonstrated to act as a molecular signal in the drought signal transduction pathway [3]. The PYL–PP2C–SnRK2 complex is the core component of the plant ABA signaling pathway [3,33]. Soybean is an important oil crop worldwide and is vital for understanding how soybean plants respond to drought stress through the ABA signaling pathway. Compared to previous studies [28], the latest version of GFF3 annotation file was used to determine *PP2C* and *SnRK2* genes of Williams 82, which would be more accurate to reveal the structure and function of the two gene families. In addition, Zhang et al. and Fan et al. both only observed expression patterns of these genes at different tissues, and neither elaborated the mechanic networks under drought stress related to the ABA signaling pathway [29,34]. In this study, we focus on the abundance of *PP2C-A* and *SnRK2* members under ABA and drought treatments. These efforts together shed new light on the evolution and functional divergence of the two gene family in soybean under drought treatment. Moreover, we ultimately cloned and isolated 22 full-length *GmSnRK2s* and 18 full-length *GmPP2C-As*.

Based on a comparison with *AtPP2C* genes, all 18 *GmPP2C-A* genes are A-type genes, which are involved in the ABA signal transduction pathway. After exogenous ABA treatment, the expression patterns of *GmSnRK2s* were altered: most of group I and II, along with several group III *GmSnRK2* genes (*GmSnRK2.1*, *GmSnRK2.4*, *GmSnRK2.6*, *GmSnRK2.8*, *GmSnRK2.10*, *GmSnRK2.21*, *GmSnRK2.22*), were significantly upregulated in response to ABA, while other expression patterns of the *GmSnRK2* genes (*GmSnRK2.2*, *GmSnRK2.3*, *GmSnRK2.5*, *GmSnRK2.11*, *GmSnRK2.12*, *GmSnRK2.13*, *GmSnRK2.15*, *GmSnRK2.17*, *GmSnRK2.18*, and *GmSnRK2.19*) varied little in response to ABA treatment (Figure 9A). Interestingly, among the ten *GmSnRK2s* that did not respond to ABA signaling, with the exception of *GmSnRK2.3*, the remaining *GmSnRK2s* had no ABREs in their promoter regions (Figure 4B), which suggested that *GmSnRK2.3* may be upregulated by other transcription factors induced by ABA signaling. Further studies should focus on elucidating the interacting proteins or upstream regulators of GmSnRK2.3 to discover a new signal transduction branch in the soybean ABA signaling pathway. Under drought treatment, most *GmSnRK2* genes could be induced by drought signals, except for *GmSnRK2.6* (Figure 9B). These results are somewhat consistent with our previous results [35] and indicate that *GmSnRK2.6* might play a role in the response to ABA treatment, independent of its role in the drought stress signaling pathway in soybean. Zhang [36] reported that *GmSnRK2.6* expression showed no difference according to a tissue-specificity expression analysis, and a possible reason is that *GmSnRK2.6* may be an ABA-induced gene and not a constitutively expressed gene.

Previous studies have indicated that multiple stress-related *cis*-elements, such as ABREs and MBSs, or these elements coupled with other abiotic or biotic *cis*-elements are required for the expression of ABA- or drought-responsive genes [27,28]. In this study, we provided a comprehensive analysis of stress-related *cis*-elements in the promoter regions of *GmPP2C-As* and *GmSnRK2s*. The results shown in Figure 3 suggest that most of the *GmPP2C-A* and *GmSnRK2* members have common ABA- and drought-related *cis*-elements, such as ABREs and MBSs; two exceptions are *GmPP2C3* and *GmPP2C15*, which do not have those *cis*-elements. Interestingly, *GmPP2C4* and *GmPP2C14* did not have MBSs but could be significantly induced in the root and leaf tissues of soybean in response to, drought treatment in our study (Figure 6B). Similar to those of *GmPP2C4* and *GmPP2C14*, the expression levels of *GmSnRK2.1*, *GmSnRK2.7* and *GmSnRK2.8*, whose promoter regions lack drought-related elements, were also significantly upregulated in response to drought signals (Figure 4B and Figure 9B). One possible explanation for these findings is that other drought-related transcription factors might directly or indirectly regulate the promoters of these genes to increase the expression of *GmPP2C4* and *GmPP2C14* via drought signaling pathway crosstalk with other hormone- or stress-related signal transduction pathways. Wei et al. [37] demonstrated that GmWRKY54 could respond to drought signals and then directly bind to the promoter of *SRK2A* to trigger ABA signal transduction to increase drought resistance. Further research could focus on identifying their upstream regulators or interacting proteins to discover novel molecular signaling mechanisms.

**Figure 11:**
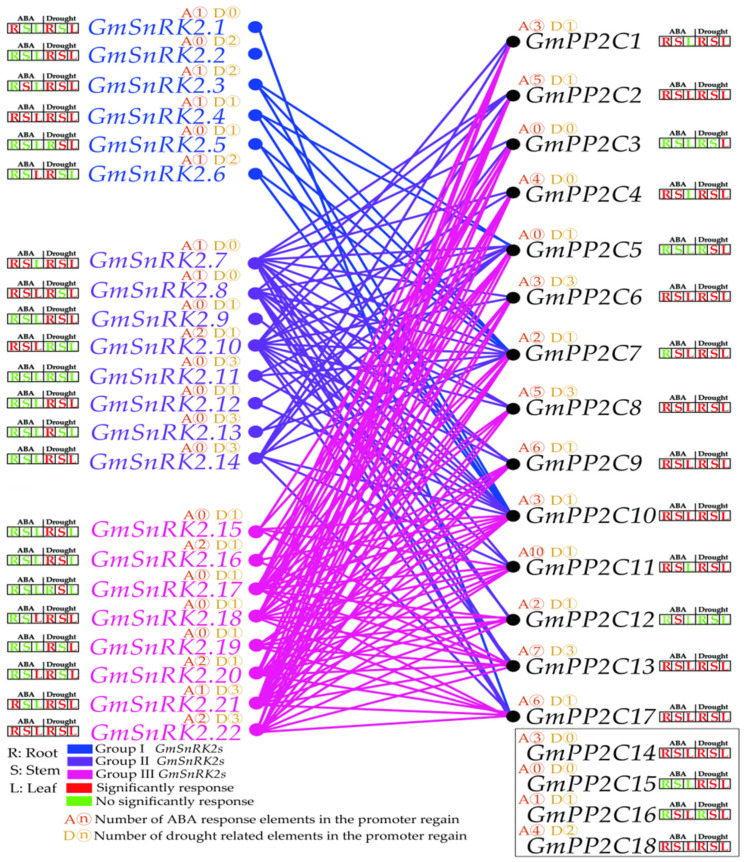
Comprehensive analysis of the expression patterns in response to ABA, drought signals and the interactions between the *GmPP2C-A* and *GmSnRK2* family members in soybean plants. The black solid line box represents the GmPP2C-As members that did not interact with GmSnRK2s, according to the Y2H assay results.

SnRK2 and PP2C proteins are known to interact to initiate the ABA signal transduction pathway [36,38]. Here, we comprehensively analyzed the expression levels of *GmPP2C-As* and *GmSnRK2s* in response to ABA and drought signaling, as well as the interactions between GmPP2C-As and GmSnRK2s in soybean (Figure 11). Except for *GmSnRK2.2* and *GmSnRK2.5*, which did not respond to ABA signals, most of the group I members of *GmSnRK2s* could respond to drought and ABA signals, which indicated that the gene functions of group I *SnRK2s* might differ between crop plant species and model plant species. The results of the expression patterns of group I and II *GmSnRK2s* in our work also support this view, and one possible reason is that *SnRK2* genes have diverse spatiotemporal expression patterns in response to ABA and drought signals in soybean. In *Arabidopsis*, *PP2C-A* genes are involved in the ABA and drought signal response network [24,29,38]. Our results were similar to those of previous studies and suggested that the functions of *GmPP2C-As* in the ABA and drought response signaling pathways may be conserved between model plant species and crop plant species. All the duplication events in *GmPP2C-As* were segmental duplication events (Figure 5A). Previous studies have demonstrated that the lack of tandem duplication events in *PP2C* family members might explain the genomic fractionation from transposon activities, relocating individual genes and driving their duplication [39,40]. Notably, although duplicate pairs of *GmSnRK2s* and *GmPP2C-As* were located on different chromosomes, we found that they exhibited similar expression patterns in response to ABA and drought signals (Figure 11). It is possible that most duplicate *GmSnRK2* and *GmPP2C-A* members have the same or similar amounts of ABA- or drought-responsive *cis*-elements in their promoter regions (Figure 5 and Figure 11). To further understand gene function, we identify miRNAs potentially targeting *GmSnRK2* and *GmPP2C-A* genes. The segmental duplicated genes (*GmPP2C3* and *GmPP2C5*) divergent from 8.3 million years ago were both targeted by Gma-miRN1266, indicating the conservation of Gma-miRN1266-mediated regulation of the duplicated genes. However, *GmPP2C2*- and *GmPP2C9*-duplicated genes divergent from 73.5 million years ago were regulated by different miRNAs, suggesting that the functional divergence may have provided genetic sources with novel biological functions during the evolution to remove function redundancy. Interestingly, our results also revealed the broad expression spectrum profile of *GmSnRK2s* and *GmPP2C-As* members, indicating that they might play different roles in the regulation of plant growth and development. Our further research will focus on the functional verification of these genes in soybean.

In this study, using a Y2H system, we analyzed the interactions between GmSnRK2s and GmPP2C-As. We found that the interactions between GmPP2C-As and GmSnRK2s are extremely complex and do not strictly comply with the functional classification of *Arabidopsis SnRK2* gene families, suggesting that the functions of *GmPP2C-As* and *GmSnRK2s* in the ABA signaling pathway in crop plant species such as soybean might differ from those in model plant species. Moreover, our results clearly showed that group III GmSnRK2s could interact with most GmPP2C-As, while group I GmSnRK2s interacted with few GmPP2C-As (Figure 11). Notably, GmSnRK2.2 neither responded to ABA signals nor interacted with any of GmPP2C-As and increased in expression in response to drought signaling, suggesting that *GmSnRK2.2* might have a distinct function in interacting with other regulatory factors involved in ABA-mediated drought signal transduction; nonetheless, further research is needed. Interestingly, most duplicated pairs of GmSnRK2s had similar GmPP2C-A gene interaction targets, which indicated that the interactions between GmSnRK2s and GmPP2CAs are highly conserved, further suggesting that these genes have essential roles (Figure 11). Our results also showed that GmPP2C5, GmPP2C7, GmPP2C10 and GmPP2C17 could interact with group I/II/III GmSnRK2 members. This suggested that they may play a central role in the crosstalk among ABA signals in response to drought stress and are involved in different signaling pathways (Figure 11). Moreover, we found that GmPP2C5 is likely to be targeted by Gma-miRN1266, which further indicated that *GmPP2C5* may be involved in regulating the mevalonate pathway and increasing resistance to plant pathogens [41,42] Future research should focus on determining whether *GmPP2C5* represents the node of the crosstalk between ABA and the drought signaling pathway and other stress-related signaling pathways. Interestingly, three group III SnRK2 members (*GmSnRK2.17*, *GmSnRK2.18* and *GmSnRK2.22*) could interact with all the tested GmPP2C-As. This suggested that they might be key regulators, that they definitively play crucial roles in regulating the drought-mediated ABA signaling pathway and that their functions may be similar to those of the *Arabidopsis* group III *SnRK2* genes, such as regulating stomatal movement and modulating soybean plant growth and development [22,23]. 

## 4. Materials and Methods

### 4.1. Plant Materials, Exogenous ABA and Drought Stress Treatments

For ABA treatment, seeds of the soybean cultivar ‘Williams 82’ were germinated in seed germination bags saturated with water. Eight days later, the seedlings were moved to a hydroponic system with half-strength Hoagland’s nutrient solution and grown in an incubator at 26 °C under a 16/8 (light/dark) photoperiod. After 21 days, the seedlings were treated with half-strength Hoagland’s nutrient solution that included 100 µM ABA (Sigma-Aldrich, Saint Louis, MO, USA) at four time points (0, 0.5, 1, 3 and 6 h), according to previous studies [43]. Seedlings treated with half-strength Hoagland’s nutrient solution were used as controls. For drought treatment, the seedlings were placed in the half-strength Hoagland’s nutrient solution with 20% PEG 6000 (26 °C; relative humidity (RH) of 50%) for six different durations (0, 0.5, 1, 3, 6 and 12 h); seedlings with half-strength Hoagland’s nutrient solution only were used as controls. All the treatments included three biological replicates. Leaves and roots were collected at each time point, immediately frozen in liquid nitrogen and stored at −80 °C until further use.

### 4.2. Gene Cloning and Sequence Analysis

Primers for isolating the full-length coding sequences of *GmPP2C-As* and *GmSnRK2s* were designed using the transcript sequence database of Williams 82 from the SoyBase website (https://www.soybase.org/). A phylogenetic tree was constructed using MEGA 5.0 with the maximum likelihood (ML) method [44]; the bootstrap values were calculated for 1000 iterations. For promoter analysis, the 1500-bp upstream sequences of the genes were scanned for *cis*-elements via PlantCARE [45]. Gene Ontology (GO) annotation analysis was performed by eggNOG-mapper and used to predict the functions of the encoded proteins [46]. The GO annotations were then plotted by ggplot2 in R. The physical and chemical characteristics of *GmPP2C-As* and *GmSnRK2s* were calculated using the ProtParam website (http://web.expasy.org/protparam/). The exon and intron structures of *GmPP2C-As* and *GmSnRK2s* were generated and visualized by ggplot2. The motifs of each identified protein were analyzed by MEME suite software (version 4.12.0; http://meme-suite.org/tools/meme/) using the following parameter: maximum number of motifs, 6–10 [47].

### 4.3. Chromosomal Location and Gene Duplication Analysis

To understand the duplication events that occurred during the evolution of *GmPP2C-As* and *GmSnRK2s*, we used MCScanX to analyze the syntenic blocks within the soybean genome based on all-vs-all BLASTP alignments [48]. The *Ka* and *Ks* values of gene pairs were calculated using an “add_ka_and_ks_to_collinearity. Pl” script. The divergence times (T) were computed as T = *Ks*/2r × 10^−6^ MYA according to the approximate substitution rate r = 6.5 × 10^−9^. The chromosomal locations of *GmPP2C-As* and *GmSnRK2s* were generated based on genomic data. All the results (including gene positions) were visualized using Circos [49].

### 4.4. Differentially Methylated Region (DMR) Detection

DMRs were determined from a previous study [50]. The methylated region for the domestication process was determined by a comparison of the methylome data of wild soybean to that of landrace populations. For the improvement process, the methylome data of the landraces were compared to those of cultivars.

### 4.5. Prediction of GmPP2C-A and GmSnRK2 Genes Targeted by miRNAs

To predict genes that might be the targets of miRNAs, psRNATarget software was firstly used to align the sequences of all *GmPP2C-A* and *GmSnRK2* members to miRNA sequences [51]. The PmiREN (https://www.pmiren.com/) was further employed to validate the miRNA–target pairs which were supported by degradome sequencing. 

### 4.6. RNA Extraction and cDNA Synthesis

Total RNA was extracted from the leaves, stems and roots using the TRIzol reagent (TransGen, Beijing, China) following the manufacturer’s procedure. The concentration and purity of the total RNA were analyzed with a NanoDrop spectrophotometer (TIANGEN, Beijing, China). cDNA was synthesized using the TransScript^®^ II One-Step gDNA Removal and cDNA Synthesis SuperMix Kit (TransGen, Beijing, China) according to the manufacturer’s protocol.

### 4.7. qPCR Analysis

The primers used for qPCR were designed by using Primer Premier 5.0 software and are listed in Supplementary Table S5. For qPCR, Cq values were acquired using an Applied Biosystems Q3 Real-Time PCR System (Thermo Fisher Scientific, Waltham, USA). The total volume of each qPCR was 25 µL. The final concentration of primer in each PCR was 0.2 µM. PCRs were performed with cDNA dilution using TB Green^TM^ Premix kit (Takara, shiga, Japan), and the means and corresponding standard errors were calculated. The qPCR conditions were as follows: 95 °C for 30 s, thirty-five PCR cycles at 95 °C for 10 s, 60 °C for 30 s, and 72 °C for 30 s. The relative quantification values were calculated using the 2^−△△Ct^ method [52], and the soybean *GmSKIP* gene was used as the internal control. For each biological replicate (three in total for each experimental condition), four technical replicates were used.

### 4.8. Y2H Assays

For the interactions between GmPP2C-As and GmSnRKs, the full-length CDS of *GmSnRK2s* was cloned into the bait vector pGBKT7 (BD), and the full-length CDS of the GmPP2Cs was cloned into the prey vector pGADT7 (AD). The GmPP2C-AD and GmSnRK2-BD constructs were then cotransformed into yeast and selected on the basis of the ability of the yeast to grow on media lacking leucine (Leu) and tryptophan (Trp). The interaction was indicated by the ability of yeast to grow on selective media that included aureobasidin A (AbA) and X–α–Gal but lacked Leu, Trp, histidine (His), and adenine (Ade). The interactions of the PacSnRK2-AD constructs with pGBKT7 were used as controls to test for yeast self-activation. The primers used to generate the various clones for the Y2H assays are listed in Supplementary Table S6.

## 5. Conclusions

Altogether, our study provides a new avenue for improving the understanding of the expression patterns of *GmPP2CAs* and *GmSnRK2s* in response to ABA and drought signals and the interactions between the two core regulatory components of the ABA signal transduction pathway in soybean plants. The results showed that the expression levels of *GmSnRK2s* and *GmPP2C-As* exhibited substantial spatiotemporal patterns. Notably, we found that the expression of *GmSnRK2s* in response to ABA and drought signals was not strictly the same as that of *Arabidopsis SnRK2* homologous genes, which indicated that the function of soybean *GmSnRK2s* may differ from that in model plant species. Moreover, our results indicated that the duplicate pairs of *GmSnRK2s* and *GmPP2C-As* have similar expression patterns, *cis*-elements and interactions, which suggested that the functions of the *GmSnRK2* and *GmPP2C-A* families were conserved after polyploidization occurred during the evolutionary history of soybean. *GmSnRK2.2* may have a distinct function in the drought-mediated ABA signaling pathway. Furthermore, the results of our Y2H assay between GmSnRK2s and GmPP2C-As revealed that *GmSnRK2.17*, *GmSnRK2.18*, *GmSnRK2.22*, *GmPP2C5*, *GmPP2C7*, *GmPP2C10* and *GmPP2C17* may play central roles in the crosstalk among ABA signals in response to drought stress. Moreover, GmPP2C5 may play multiple roles in the crosstalk between ABA and drought signals and other stress signals. Taken together, our results provide worthwhile information for understanding the expression and interaction network of the *GmSnRK2* and *GmPP2C-A* families. These findings also provide a scientific foundation for improving and breeding new varieties of soybean to adapt to drought stress conditions.

## Data Availability

Not applicable.

## Supplementary Materials

The following supporting information can be downloaded at: https://www.mdpi.com/article/10.3390/ijms232113166/s1.

## Abbreviations

ABA	Abscisic acid
PYLs	PYR/PYL/RCAR ABA receptors
SnRK2	Class III SNF–1–related protein kinase 2
PP2C-As	Group A Type 2C protein phosphatase
ATP	Adenosine triphosphate
TIS	Transcription initiation site
MW	Molecular weight
kDa	Kilodalton
GRAVY	Grand average of hydropathy
UTR	Untranslated regions
ROS	Reactive oxygen species
GA	Gibberellin
IAA	Indole–3–acetic acid
AbA	Aureobasidin A
Leu	Leucine
Trp	Tryptophan
His	Histidine
Ade	Adenine
MYA	Million years ago
MBS	MYB–binding site involved in drought-inducibility
ABF	ABA response element-binding factors
ABRE	ABA responsive elements
ABI	ABA INSENSITIVE transcription factor
AREB	ABA-responsive element binding protein
ARE	Anaerobic induce element
HSE	Heat stress response element
ERE	Ethylene response element
GARE	Gibberellin response element
LTR	Low-temperature response element
WUN-motif	Wound-responsive element
O_2_-site	*cis*-acting regulatory element involved in zein metabolism regulation
TC-rich repeats	Defense and stress response element
CGTCA-motif	MeJA response element
P-box	Gibberellin response element
Box-W1	Fungal elicitor response element
CE3	ABA and VP1 response element
CCAAT-box	MYBHv1 binding site
qPCR	Quantitative real-time polymerase chain reaction
MeJA	Methyl jasmonic acid
JA	Jasmonic acid
SA	Salicylic acid
PI	Isoelectric point
Bp	Base pair
Y2H	Yeast two hybrid
GO	Gene ontology
DMR	Differentially methylated region
Dos-DMR	Process of soybean domestication with differentially methylated region
Imp-DMR	In the improvement process with differentially methylated region
ML	Maximum Likelihood
