# Supplementary material for: Genome-Wide Identification, Expression and Interaction Analysis of GmSnRK2 and Type A PP2C Genes in Response to Abscisic Acid Treatment and Drought Stress in Soybean Plant
Source: Int J Mol Sci. 2022 Oct 29;23(21):13166. doi: 10.3390/ijms232113166 (PMC9653956; doi:10.3390/ijms232113166)
Supplement: Supplementary file 1 [file ijms-23-13166-s001.zip › Table S1.pdf]

**Table S1 Characteristics of *GmPP2Cs-As* in soybean**							
**Gene name**	**Gene ID**	**Amino acid**	**MW (Da)**	**pI**	**Instability index**	**GRAVY**	**Chromosomes**
GmPP2C1	Glyma.01g225100	324	35,093.05	6.75	47.55	-0.228	1
GmPP2C2	Glyma.02g250200	407	44,933.13	6.67	53.82	-0.361	2
GmPP2C3	Glyma.04g053800	544	59,216.90	4.86	43.36	-0.216	4
GmPP2C4	Glyma.05g227100	384	42,155.30	5.36	67.38	-0.348	5
GmPP2C5	Glyma.06g054000	544	58,934.53	4.83	45.69	-0.182	6
GmPP2C6	Glyma.08g033800	239	26,871.69	6.05	67.40	-0.292	8
GmPP2C7	Glyma.09g066500	522	57,118.04	5.42	42.20	-0.545	9
GmPP2C8	Glyma.11g018000	246	26,603.24	7.55	46.09	-0.282	11
GmPP2C9	Glyma.11g222600	401	43,834.33	5.51	56.39	-0.438	11
GmPP2C10	Glyma.13g106800	536	58,563.88	5.07	47.95	-0.386	13
GmPP2C11	Glyma.14g066400	296	32,716.15	6.52	53.59	-0.391	14
GmPP2C12	Glyma.14g108400	557	60,815.60	4.62	41.13	-2.05	14
GmPP2C13	Glyma.14g162100	283	30,896.06	8.04	50.00	-0.297	14
GmPP2C14	Glyma.15g172500	527	57,849.70	5.22	44.75	-0.541	15
GmPP2C15	Glyma.17g052500	400	43,093.21	4.66	44.33	-0.288	17
GmPP2C16	Glyma.17g218700	465	50,408.78	4.52	37.34	-0.069	17
GmPP2C17	Glyma18g035000	400	44,067.66	5.17	58.5	-0.443	18
GmPP2C18	Glyma.19g069200	301	32,503.45	6.68	56.32	-0.456	19
