# Supplementary material for: Genome-Wide Identification, Expression and Interaction Analysis of GmSnRK2 and Type A PP2C Genes in Response to Abscisic Acid Treatment and Drought Stress in Soybean Plant
Source: Int J Mol Sci. 2022 Oct 29;23(21):13166. doi: 10.3390/ijms232113166 (PMC9653956; doi:10.3390/ijms232113166)
Supplement: Supplementary file 1 [file ijms-23-13166-s001.zip › Table S2.pdf]

**Table S2 Characteristics of *GmSnRK2s* in soybean**							
**Gene name**	**Gene ID**	**Amino acid residues**	**MW (Da)**	**pI**	**instability index**	**GRAVY**	**Chromosome**
GmSnRK2.1	Glyma.04g205400	349	40,349.00	6.10	45.81	-0.497	4
GmSnRK2.2	Glyma.06g160100	346	39,979.67	6.48	43.51	-0.468	6
GmSnRK2.3	Glyma.05g197700	351	40,428.98	5.95	44.26	-0.523	5
GmSnRK2.4	Glyma.08g005100	351	40,386.86	5.75	46.31	-0.529	8
GmSnRK2.5	Glyma.08g188300	352	40,262.80	6.10	49.33	-0.486	8
GmSnRK2.6	Glyma.12g169800	359	41,081.59	5.82	48.44	-0.537	12
GmSnRK2.7	Glyma.02g208500	338	38,301.73	5.38	31.82	-0.267	2
GmSnRK2.8	Glyma.14g176700	338	38,176.66	5.38	37.08	-0.251	14
GmSnRK2.9	Glyma.05g176100	227	25,639.95	4.58	53.31	-0.349	5
GmSnRK2.10	Glyma.08g133600	345	39,236.66	5.15	41.37	-0.300	8
GmSnRK2.11	Glyma.05g066700	336	38,124.45	5.60	37.51	-0.447	5
GmSnRK2.12	Glyma.17g148800	287	32,698.73	8.33	32.29	-0.183	17
GmSnRK2.13	Glyma.01g204200	339	38,559.92	6.44	39.70	-0.432	1
GmSnRK2.14	Glyma.11g038800	339	38,660.03	6.39	37.40	-0.450	11
GmSnRK2.15	Glyma.02g135500	264	29,816.59	4.69	46.23	-0.358	2
GmSnRK2.16	Glyma.07g209400	354	40,197.60	5.04	44.90	-0.353	7
GmSnRK2.17	Glyma.07g178600	205	23,309.80	6.71	41.82	-0.319	7
GmSnRK2.18	Glyma.20g009600	315	36,032.92	4.94	47.58	-0.303	20
GmSnRK2.19	Glyma.01g183500	313	35,323.05	4.78	39.87	-0.217	1
GmSnRK2.20	Glyma.11g058800	267	30,325.90	6.31	36.16	-0.196	11
GmSnRK2.21	Glyma.05g081900	360	40,801.37	4.75	43.41	-0.268	5
GmSnRK2.22	Glyma.17g178800	293	33,184.09	6.25	39.95	-0.218	17
