# Supplementary material for: Genome-Wide Identification, Expression and Interaction Analysis of GmSnRK2 and Type A PP2C Genes in Response to Abscisic Acid Treatment and Drought Stress in Soybean Plant
Source: Int J Mol Sci. 2022 Oct 29;23(21):13166. doi: 10.3390/ijms232113166 (PMC9653956; doi:10.3390/ijms232113166)
Supplement: Supplementary file 1 [file ijms-23-13166-s001.zip › Table S3.pdf]

**Table S3. Segmental duplicated gene pairs of *GmPP2C-As* and *GmSnRK2s* genes**					
**Duplicated gene pairs**		***Ka***	***Ks***	***Ka/Ks***	**Time (MYA)**
GmPP2C1	*GmPP2C8*	0.098621	0.186471	0.528881	14.34395
GmPP2C2	*GmPP2C9*	0.223641	0.955546	0.234045	73.50352
GmPP2C2	*GmPP2C11*	0.058882	0.230327	0.255646	17.71743
GmPP2C2	*GmPP2C17*	0.20438	0.84824	0.240946	65.24926
GmPP2C3	*GmPP2C5*	0.028138	0.108004	0.260526	8.308018
GmPP2C4	*GmPP2C6*	0.027845	0.097286	0.286221	7.483572
GmPP2C7	*GmPP2C14*	0.04644	0.118849	0.390746	9.142246
GmPP2C9	*GmPP2C11*	0.203821	0.999547	0.203914	76.88823
GmPP2C9	*GmPP2C17*	0.040155	0.209889	0.191318	16.1453
GmPP2C11	*GmPP2C17*	0.210302	1.04264	0.201702	80.20309

MYA: million years ago
