# Supplementary material for: Genome-Wide Identification, Expression and Interaction Analysis of GmSnRK2 and Type A PP2C Genes in Response to Abscisic Acid Treatment and Drought Stress in Soybean Plant
Source: Int J Mol Sci. 2022 Oct 29;23(21):13166. doi: 10.3390/ijms232113166 (PMC9653956; doi:10.3390/ijms232113166)
Supplement: Supplementary file 1 [file ijms-23-13166-s001.zip › Table S4.pdf]

Table S4. Segmental duplicated gene pairs of *GmSnRK2s* genes

Duplicated gene pairs		*Ka*	*Ks*	*Ka*/*Ks*	Time(MYA)
GmSnRK2.19	*GmSnRK2.21*	0.073144495	0.603619293	0.121177	46.43225
GmSnRK2.19	*GmSnRK2.20*	0.002383792	0.0755075	0.03157	5.808269
GmSnRK2.19	*GmSnRK2.22*	0.069934678	0.550542238	0.127029	42.3494
GmSnRK2.7	*GmSnRK2.8*	0.016690536	0.079112406	0.210972	6.08557
GmSnRK2.1	*GmSnRK2.3*	0.033996588	0.380283676	0.089398	29.25259
GmSnRK2.1	*GmSnRK2.2*	0.002491438	0.100492152	0.024792	7.730166
GmSnRK2.1	*GmSnRK2.4*	0.03431148	0.393136061	0.087276	30.24124
GmSnRK2.3	*GmSnRK2.2*	0.035679304	0.402383359	0.08867	30.95257
GmSnRK2.9	*GmSnRK2.10*	0.011393526	0.08684389	0.131195	6.680299
GmSnRK2.3	*GmSnRK2.4*	0.006137514	0.071376259	0.085988	5.490481
GmSnRK2.11	*GmSnRK2.14*	0.123880319	0.508638231	0.243553	39.12602
GmSnRK2.21	*GmSnRK2.20*	0.073098563	0.586938029	0.124542	45.14908
GmSnRK2.21	*GmSnRK2.22*	0.005976723	0.116959236	0.051101	8.996864
GmSnRK2.2	*GmSnRK2.4*	0.037304469	0.393687819	0.094756	30.28368
GmSnRK2.16	*GmSnRK2.18*	0.033542491	0.422144377	0.079457	32.47264
GmSnRK2.20	*GmSnRK2.22*	0.069890809	0.534942919	0.130651	41.14946

MYA, million years ago
