# Supplementary material for: Genome-Wide Identification, Expression and Interaction Analysis of GmSnRK2 and Type A PP2C Genes in Response to Abscisic Acid Treatment and Drought Stress in Soybean Plant
Source: Int J Mol Sci. 2022 Oct 29;23(21):13166. doi: 10.3390/ijms232113166 (PMC9653956; doi:10.3390/ijms232113166)
Supplement: Supplementary file 1 [file ijms-23-13166-s001.zip › Table S5.pdf]

**Table S5. Primers used for QPCR assays of type A *GmPP2C* and *GmSnRK2* genes**			
Gene name	Forward primer (5′ to 3′)	Reverse primer (5′ to 3′)	Product (bp)
GmPP2C1 (Glyma.01g225100)	CATCCCACTCAACACTACTACAACT	TACCCATCCTAACAATACAAGACAA	165
GmPP2C2 (Glyma.02g250200)	CTAGAGTGCTTGGAGTGTTGGCT	ATTTTTCTTCCCTTTTTTTGTCA	489
GmPP2C3 (Glyma.04g053800)	ATTTTTATTTTTGGTCATTTTCTTC	CTCTGTCTACCTCCGTCAACTCTAG	251
GmPP2C4 (Glyma.05g227100)	CAACTACGACACCAGTACCGTTG	TCTCTTTCCTTCTCCCTATCACC	337
GmPP2C5 (Glyma.06g054000)	ATCTGTCCAACTCCATTGTCTTCT	CTCGTTTCTTCCTTCTCTTCTTTT	266
GmPP2C6 (Glyma.08g033800)	ACTTCAGCTTGTGTTTCTCCTA	CCAGTTGTCACTTGTCATGAAG	80
GmPP2C7 (Glyma.09g066500)	CCTAACCAAACTGACCAACTAAAAAA	CACTCAACTCTCAACCTATGAAAACA	238
GmPP2C8 (Glyma.11g018000)	ACGCACGAAAAGAGTCAGATGA	TTGGAAAATTAGACGCGAAAAG	302
GmPP2C9 (Glyma.11g222600)	TTACTTAGGTTCTGAATGGCTGG	TCGTTTCGGAAATATTGTCGTGG	289
GmPP2C10 (Glyma.13g106800)	TCCCCCTTGACTCCGTTTTAG	TTTTTTTCCACCCTTTTCCCT	251
GmPP2C11 (Glyma.14g066400)	CCCCACTTGCTTTTCATTTTTTT	TACCCCATACCTCGGATACTCCT	433
GmPP2C12 (Glyma.14g108400)	TGTGTTTAGAGATATGGGGCGT	AAGGTGGTGATAGTGAAATGGG	298
GmPP2C13 (Glyma.14g162100)	CACAACATGCCGTTTCTCTTTTA	ATCTCCTTCCTGCTCCCTATCAC	491
GmPP2C14 (Glyma.15g172500)	AAGCAAAGAAATCTGTGAAGAAAAT	AAAAAAAAAGGAATAAAAAGGAGGA	131
GmPP2C15 (Glyma.17g052500)	TATTTGCCTTTGGTTTGCTATGA	ACTTTTTTCTCCACCCTTTTCCC	251
GmPP2C16 (Glyma.17g218700)	CTCAAAGAAAATATAAGAGCAAG	AGAGGAGAATCAGGTAACAGTAC	418
GmPP2C17 (Glyma.18g035000)	CATAGTTCGGATAATGTGAGCGT	GATTCCTTAGTTTGGTTTGGTTT	129
GmPP2C18 (Glyma.19g069200)	ATGTCGTCATGTTCTGAGTTTCG	TTTTGGCGTCTTCATAGTCGTTT	103
GmSnRK2.1 (Glyma.04g205400)	GGGTCATCCTGGTCCTCAAA	TCATCTTTGCTGCACTCACG	173
GmSnRK2.2 (Glyma.06g160100)	ATCATCCTGGTCCTCAAAGGG	CTGCATTCGCGACCGAAATC	162
GmSnRK2.3 (Glyma.05g197700)	GCCTAGATACTGGAGGAGGG	TGTAGCAAATCCATTAAGGAGAATA	200
GmSnRK2.4 (Glyma.08g005100)	CCTCATCACCACGCACAAC	GGCTGAAAGAAGGGGTTACG	152
GmSnRK2.5 (Glyma.08g188300)	TCATCTTCTTCAGTCTCAGCCA	GCTCAAGCTGCCTACTACAG	191
GmSnRK2.6 (Glyma.12g169800)	CTTCTTCAGCCTCGGTCACT	TTGTTGAGGAGGCCAAGACT	138
GmSnRK2.7 (Glyma.02g208500)	TCAGCATCAAAGTCATCCAGG	GCCCATGGAACTGACGGA	174
GmSnRK2.8 (Glyma.14g176700)	GATCTTCATCAGCATCCAAGTCA	GAACTTGCCCATAGAACAGATG	188
GmSnRK2.9 (Glyma.05g176100)	GCACAGACAAAATCACCGCTT	TGCAAAATGATGATGTGAATGACG	199
GmSnRK2.10 (Glyma.08g133600)	GCAAGATGAGTTGGAGTAAGC	CAGGAAACTTTGGTGTGGCA	187
GmSnRK2.11 (Glyma.05g066700)	GTCCAACTTCTCCTGCTTTGG	CCAAGAGGATCACCATCCCG	198
GmSnRK2.12 (Glyma.17g148800)	GAATTGAAGATACATACCTTGGCA	TGGTGCATACCCATTTGAAGAT	174
GmSnRK2.13 (Glyma.01g204200)	CGTAGTCGGGTATGGCGTAT	ATGTTTGGTCGTGTGGTGTG	135
GmSnRK2.14 (Glyma.11g038800)	CCTCATCACCACGCACAAC	GGCTGAAAGAAGGGGTTACG	152
GmSnRK2.15 (Glyma.02g135500)	CAGCAACAAGTTCCTCAGTATGTT	TGTTGGGCCAGGAATGGATTT	131
GmSnRK2.16 (Glyma.07g209400)	TCTATCTGCTTTTCACCTCTCTCA	CCGGGAATGGATTTGCCGAT	158
GmSnRK2.17 (Glyma.07g178600)	TCGTCATCAATGTCCAAGCTA	GAACACGATGAACAGGCAA	150
GmSnRK2.18 (Glyma.20g009600)	GCCAAATGTGTAGGGGTCAA	CCGTGATATTGGGTCTGGGA	201
GmSnRK2.19 (Glyma.01g183500)	GGTCCCCGCAGCTGGTATG	TGACCCTGCAGAGAGAATCACTATT	187
GmSnRK2.20 (Glyma.11g058800)	GTCCCTGCAGCGGGTAT	TGCAGAGAGAATCACTATTCCTGA	180
GmSnRK2.21 (Glyma.05g081900)	CTTTAAACGAGGTGCCGAGC	ACTGAGGATGAGGCTCGATT	135
GmSnRK2.22 (Glyma.17g178800)	ATCCTCGGTAAAACGCCCA	ATAGAGGAGGGGCAATCACATT	152

GmSKIP	GAGCCCAAGACATTGCGAGAG	CGGAAGCGAAGAACTGAACC	
