# Supplementary material for: Genome-Wide Identification, Expression and Interaction Analysis of GmSnRK2 and Type A PP2C Genes in Response to Abscisic Acid Treatment and Drought Stress in Soybean Plant
Source: Int J Mol Sci. 2022 Oct 29;23(21):13166. doi: 10.3390/ijms232113166 (PMC9653956; doi:10.3390/ijms232113166)
Supplement: Supplementary file 1 [file ijms-23-13166-s001.zip › Table S6.pdf]

Table S6. Primers used for the yeast two-hybrid assay (bases underlined represent restriction enzyme sites)

Gene name	Froward primer 5′→3′	Reverse primer 5′→3′
GmPP2C1-AD	GAATTCATGTGCATGCCCATACGTGAT	GGATCCTCAAGTTGTAGTAGTGTTGAGTGGGA
GmPP2C2-AD	GAATTCATGGCTGGAATTTGCTGTGGA	GAGCTCTTAATTAATTCCTTTTGTTGTTGCCAT
GmPP2C3-AD	GAATTCATGGAGGAGATGTCAACCACG	GAGCTCTCAAGTCTTGCTCTTAAATTTTCTTT
GmPP2C4-AD	GAATTCATGGACGAGGACGAGCAACT	GAGCTCTTAGGAAATTAAAGGAGGCCTTTG
GmPP2C5-AD	GAATTCATGGAGGAGATGTCAACCACG	GAGCTCTCATGTCTTGCTCTTAAATTTCCTTT
GmPP2C6-AD	GAATTCATGGACGAGGACGAGCAACT	GGATCCTTAGGAAATTAAAGGAGGCCTTTG
GmPP2C7-AD	GAATTCATGGAGGAAATAACTTCGACTGTTG	GAGCTCTTATTCTTTTTTCTTGATTTTTCTCTGAGG
GmPP2C8-AD	GAATTCATGCATTCGACGAAGAGTAGCAC	GGATCCTCAAGTTGTAGTAGTGTTGAGTTGGATG
GmPP2C9-AD	GAATTCATGGCTGGAATTTGCTGTGGT	CCCGGGTTAATTGGAGTTTGATGATTGTCGTT
GmPP2C10-AD	GAATTCATGGAGGAAATATCTTCTAGTGTTGC	CCCGGGTTATGGCTTTCTCTTAATTTTTCTTTGA
GmPP2C11-AD	GAATTCATGGCTGGGATTTGCTGTGG	GAGCTCTTAGGTTCTCCTCAAATCAACCACC
GmPP2C12-AD	GAATTCATGGAGGAGATGTCCTTTATTGTTG	GAGCTCTCATGTCTTGCTCTTATATTTTCTATAGGG
GmPP2C13-AD	GAATTCATGTTCAGTTCGTTGCATGCAA	GGATCCTCAATCAGTTGTTACTGATCCTCTTAG
GmPP2C14-AD	GAATTCATGGAGGAAATAACTTCGACGG	GGATCCTTATTCTTTTTTCTTGATTTTTCTCTGAG
GmPP2C15-AD	GAATTCATGGAGGAAATATCTTCTAGTGTTGC	GGATCCTTATGGCTTTCTCTTAATTTTTCTTTG
GmPP2C16-AD	GAATTCATGGAGGAGATGTCCTTTGCG	GGATCCTCATGTCTTGCTCTTATATTTTCTTTG
GmPP2C17-AD	GAATTCATGGCTGGAATTTGCTGTGGT	CCCGGGTTAATTAACGTCGTTGTAGTTTGATGAT
GmPP2C18-AD	GAATTCATGAAGACGCCAAAACGCC	GGATCCTCAATCAGTTGTTACTGTTCCTCTTAGC
GmSnRK2.1-BD	GAATTCATGGATAAGTACGAGGCTGTTAAGG	GTCGACTTAACTAATTTGAAATTCGCCACTT
GmSnRK2.2-BD	GAATTCATGGATAAGTACGAGACCGTTAAGG	GTCGACTTAACTAATTTGAAATTCGCCACTT
GmSnRK2.3-BD	GAATTCATGGATAAGTATGAGGCTGTCAAGG	GTCGACTTAACTGATTTGAAATTCTCCACTTG
GmSnRK2.4-BD	GAATTCATGGATAAGTATGAGGCTGTCAAGG	GTCGACTTAACTGATTTGAAATTCTCCACTTG
GmSnRK2.5-BD	GAATTCATGGAGAAATACGAGCTCGTGAA	GTCGACTTAACTGACATGAAATTCCCCACTT
GmSnRK2.6-BD	GAATTCATGGACAAATACGAGCTCGTCAA	GTCGACTTAACTGACATGAAATTCCCCACTT
GmSnRK2.7-BD	GAATTCATGGAGCGCTATGAGATCCTCA	GTCGACTTAAATAGGACACACAAATTCACCACT
GmSnRK2.8-BD	GAATTCATGGAGGGCTATGAGATCCTCA	GTCGACTTAAATAGGACACACAAATTCACCACT
GmSnRK2.9-BD	GGGATCCGTATGCTTGCAAGCTTAACCATGG	CTGCAGTCACAATGCACAGACAAAATCACC
GmSnRK2.10-BD	GGGATCCGTATGGAACGGTATGAGATAATTAAAGATATT	CTGCAGTCACAATGCACAGACAAAATCACC
GmSnRK2.11-BD	GAATTCATGGAGGAACGGTACGAGCC	GTCGACTCACACTTGTTCATAGTCACCACTAAC
GmSnRK2.12-BD	GAATTCATGGAAGAACGGTATGAGCCAT	GTCGACTCACACTTGTTCATAGTCACCACTAAC
GmSnRK2.13-BD	GAATTCATGGAAGAACGGTACGAGACTTTG	GTCGACCTAATTAAGGGCTAGGTATTTGGCATA
GmSnRK2.14-BD	GAATTCATGGACGAACGGTACGAGACTT	GTCGACTTAATCAAGGGTTAGGTATTTGGC
GmSnRK2.15-BD	GAATTCATGGATTTGCCGATCATGCA	GTCGACTTACATAGCATACACAATTTCTCCACT
GmSnRK2.16-BD	GAATTCATGAGTGTTGGGCCGGGAA	GTCGACTTACATAGCATACACAATTTCTCCACT
GmSnRK2.17-BD	GAATTCATGGATCGTTCTGCTATGACTGTT	GTCGACTCATATTGCATAAACTATTTCTCCACTG
GmSnRK2.18-BD	GAATTCATGGATCGTTCTGCTATGACTGTT	GTCGACTTACATTGCATAAACTATTTCTCCACTG
GmSnRK2.19-BD	GAATTCATGGATCGGCCGGCGACCG	GTCGACTTATATGGCATACACTATCTCCCCACTG
GmSnRK2.20-BD	GAATTCATGGATCGGCCGGCGACCG	GTCGACTTATATGGCATACACTATCTCCCCACTG
GmSnRK2.21-BD	GAATTCATGGATCGGGCGGCGTTGA	GTCGACCTATATGGCATACACTATTTCCCCACTG
GmSnRK2.22-BD	GAATTCATGGATCGGGCGGCGTTGAC	GTCGACCTATATGGCATACACTATCTCCCCACTG
