# Supplementary figures and images for: Genome-Wide Identification, Expression and Interaction Analysis of GmSnRK2 and Type A PP2C Genes in Response to Abscisic Acid Treatment and Drought Stress in Soybean Plant
Source: Int J Mol Sci. 2022 Oct 29;23(21):13166. doi: 10.3390/ijms232113166 (PMC9653956; doi:10.3390/ijms232113166)

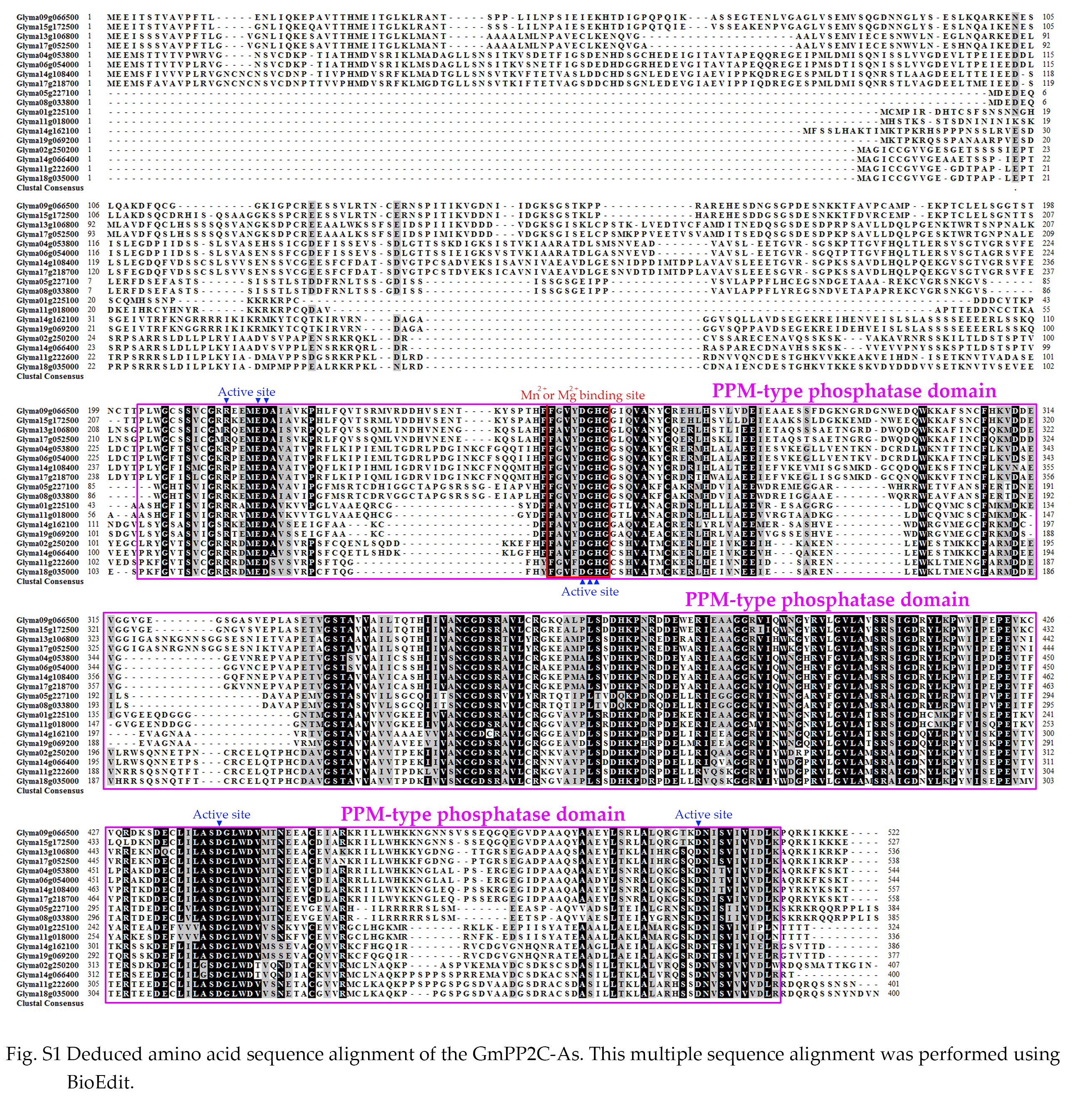

Supplement: Supplementary file 1 [file ijms-23-13166-s001.zip › Fig S1.tif]

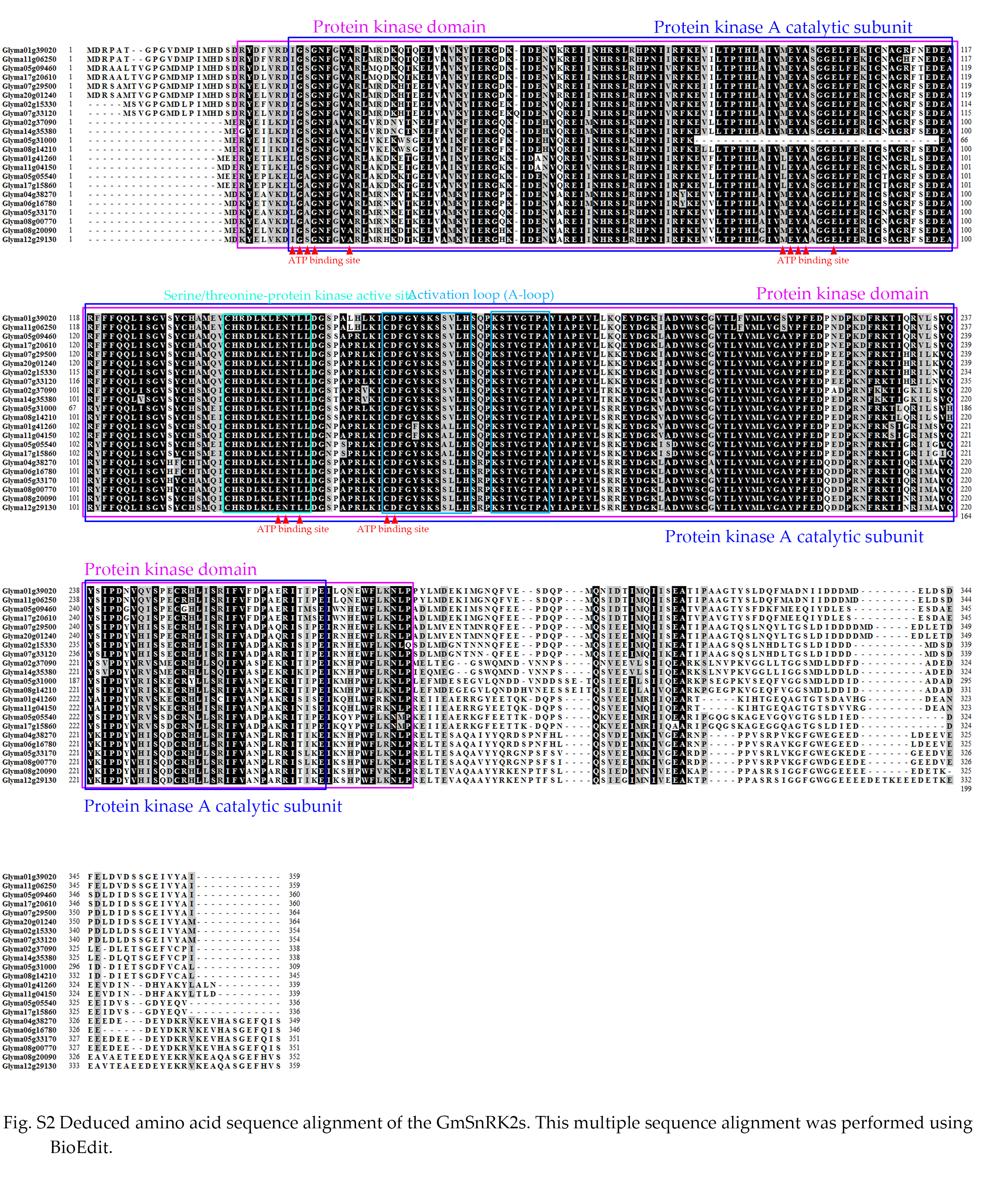

Supplement: Supplementary file 1 [file ijms-23-13166-s001.zip › Fig S2.tif]
